# SARS-CoV-2 and the pandemic of COVID-19

**DOI:** 10.1136/postgradmedj-2020-138386

**Published:** 2020-08-11

**Authors:** Md Tanveer Adil, Rumana Rahman, Douglas Whitelaw, Vigyan Jain, Omer Al-Taan, Farhan Rashid, Aruna Munasinghe, Periyathambi Jambulingam

**Affiliations:** Department of Upper GI and Bariatric Surgery, Luton and Dunstable University Hospital, Luton LU4 0DZ, UK; Department of Gynaecology & Obstetrics, Calderdale Royal Hospital, Halifax HX3 0PW, UK; Department of Upper GI and Bariatric Surgery, Luton and Dunstable University Hospital, Luton LU4 0DZ, UK; Department of Upper GI and Bariatric Surgery, Luton and Dunstable University Hospital, Luton LU4 0DZ, UK; Department of Upper GI and Bariatric Surgery, Luton and Dunstable University Hospital, Luton LU4 0DZ, UK; Department of Upper GI and Bariatric Surgery, Luton and Dunstable University Hospital, Luton LU4 0DZ, UK; Department of Upper GI and Bariatric Surgery, Luton and Dunstable University Hospital, Luton LU4 0DZ, UK; Department of Upper GI and Bariatric Surgery, Luton and Dunstable University Hospital, Luton LU4 0DZ, UK

**Keywords:** SARS-CoV-2, COVID-19, viral infections, global health, Coronavirus

## Abstract

SARS-CoV-2 is a virus that is the cause of a serious life-threatening disease known as COVID-19. It was first noted to have occurred in Wuhan, China in November 2019 and the WHO reported the first case on December 31, 2019. The outbreak was declared a global pandemic on March 11, 2020 and by May 30, 2020, a total of 5 899 866 positive cases were registered including 364 891 deaths. SARS-CoV-2 primarily targets the lung and enters the body through ACE2 receptors. Typical symptoms of COVID-19 include fever, cough, shortness of breath and fatigue, yet some atypical symptoms like loss of smell and taste have also been described. 20% require hospital admission due to severe disease, a third of whom need intensive support. Treatment is primarily supportive, however, prognosis is dismal in those who need invasive ventilation. Trials are ongoing to discover effective vaccines and drugs to combat the disease. Preventive strategies aim at reducing the transmission of disease by contact tracing, washing of hands, use of face masks and government-led lockdown of unnecessary activities to reduce the risk of transmission.

## INTRODUCTION

Severe acute respiratory syndrome coronavirus 2 (SARS-CoV-2) is a novel coronavirus (CoV) previously unknown to mankind. It is classified as a beta-CoV of group 2B and is the cause of a serious life-threatening disease known as coronavirus disease of 2019 (COVID-19).^1^

### Estimation of the problem

**Figure 1:**
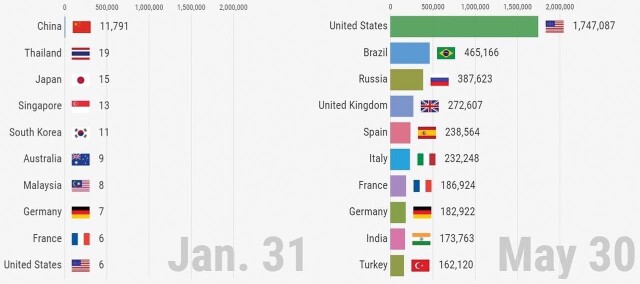
Total number of new cases at the beginning of the pandemic (January 31, 2020) and at the peak and beyond (May 30, 2020) due to COVID-19 shown in the 10 most effected countries.^5^

**Figure 2:**
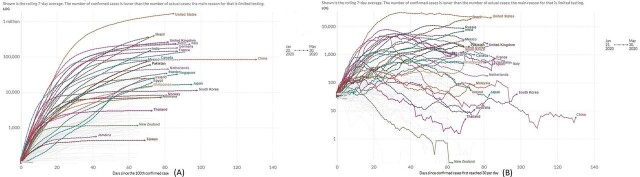
Total confirmed new cases (A) and daily new confirmed cases (B) due to COVID-19 from the beginning of the pandemic until May 30, 2020 in different countries. Some data points are interpolated to account for missing values. *Sources: Local governments; The Centre for Systems Science and Engineering at John Hopkins University; National Health Commission of the People’s Republic of China; World Health Organization*.^6^

**Figure 3:**
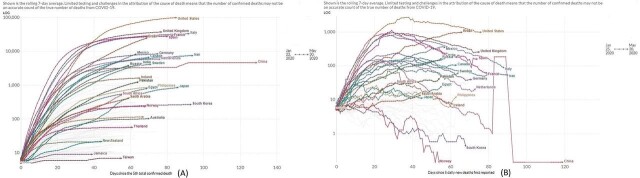
Total confirmed new deaths (A) and daily new confirmed deaths (B) due to COVID-19 from the beginning of the pandemic until May 30, 2020 in different countries. Some data points are interpolated to account for missing values. *Sources: Local governments; The Centre for Systems Science and Engineering at John Hopkins University; National Health Commission of the People’s Republic of China; World Health Organization.* ^6^

The evolution of our understanding of SARS-CoV-2 has been studded with as much controversy and mystery as the disease it causes. It was first noted to have occurred in Wuhan, China as the first case was reported by the WHO on December 31, 2019.^1^ However, some experts believe that the earliest case of COVID-19 was detected as early as November 17, 2019.^2^ Subsequently, COVID-19 has spread rapidly throughout the world and has reached pandemic proportions affecting all continents. WHO declared the outbreak a Public Health Emergency of International Concern on January 30, 2020 and on March 11, 2020, the outbreak was declared a global pandemic.^3^ The European Centre of Disease Prevention and Control has estimated a total of 5 899 866 cases of COVID-19 between December 31, 2019 and May 30, 2020 in accordance with the applied case definitions and testing strategies in the affected countries, including 364 891 deaths due to the disease.^4^ Total confirmed new cases and daily confirmed new cases until May 30, 2020 due to the COVID-19 pandemic are tabulated in figures 1 and 2.^5 6^  Figure 3 shows the total confirmed new deaths and daily confirmed new deaths due to COVID-19 until May 30, 2020.^6^

## AETIOLOGY OF COVID-19

It has been widely speculated that the Wuhan’s animal market is responsible for the first human transmission of SARS-CoV-2.^1 2^ The earliest genomic identification and characterisation of SARS-CoV-2 responsible for the epidemic in Wuhan, China was done from virions sampled from five patients.^7^ The sequences were found to share 79.6% similarity to SARS-CoV that caused the severe acute respiratory syndrome (SARS) outbreak in 2002.^7^ It also showed 50% homology to MERS-CoV sequences that was responsible for the epidemic of Middle-eastern respiratory syndrome (MERS) in the Middle East in 2012–2013.^8^ However, genetic analysis of SARS-CoV-2 also showed a 96.2% similarity at whole-genome level to a bat Coronavirus, BatCoV RaTG13.^7^ This similarity has led to the suggestion that the transmission of bat CoV to humans could possibly have been aided with the help of an intermediate host that could act as a host for CoVs. Pangolins have been identified as a natural reservoir of SARS-CoV-2 like CoV. According to one study, pangolin-CoV was found to be 91.02% identical to SARS-CoV-2 at whole-genome level, being the second closest relative of human SARS-CoV-2 behind BatCoV RaTG13.^9^ On October 24, 2019, lung samples of two dead Malayan pangolins with frothy liquid in their lungs and pulmonary fibrosis detected the existence CoV that showed 80.24%–88.93% similarity to known SARS-CoVs at genomic level.^10^ The findings of this study coincided in timing with the COVID-19 epidemic in Wuhan, China, which, along with the fact that genomic analysis of human SARS-CoV-2 show greater similarity to the Pangolin-CoV than other SARS-CoVs lends support to the hypothesis of pangolins acting as an intermediate host in the transimission of SARS-CoV-2 from bats to humans. However, another study identified a unique peptide insertion in human SARS-CoV-2 that could be responsible for proteolytic cleavage of spike protein imparting host range and transmissibility which was absent in pangolin-CoV, dismissing the hypothesis of COVID-19 outbreak coming from pangolins.^11^ The possibility of a wide range of other animals like cats, cows, buffaloes, goats, sheeps and pigeons have also been suggested to act as an intermediate host in the transmission of SARS-CoV-2 from bats to humans.^12^

Another unconfirmed hypothesis that has received mixed response is the possibility of the virusoriginating in Wuhan’s Centre of Disease Control and Prevention, located just 300 yards away from Wuhan’s animal market or the Wuhan Institute of Virology located eight miles away from the animal market.^13^ Conspiracy theories about a possible accidental leak from either of these laboratories known to be experimenting with bats and bat CoVs that has shown some structural similarity to human SARS-CoV-2 has been suggested, but largely dismissed by most authorities. It has also been debated that the national mortality figures in China due to COVID-19 may have been under-reported.^14^ According to a study published in *Nature Medicine*, molecular analysis of SARS-CoV-2 suggests that it has probably spread to humans as a result of natural selection rather than being a laboratory construct or a purposely manipulated virus.^15^

## PATHOGENESIS AND DIAGNOSIS OF COVID-19

### Entry and replication

It has been reasoned that lung epithelium is the primary target of the virus. The receptor-binding domain (RBD) of SARS-CoV-2 S-protein determines the entry of the virus into human host cells through the Angiotensin converting enzyme 2 (ACE2) receptor, and this mechanism is similar to the original SARS-CoV which shares significant homology in the RBD of S-protein.^16^ Speculations are that genetic variations in ACE2 gene and variability in the degree of its expression between individuals and ethnicities could explain the variation observed in clinical intensity and virulence of COVID-19 infection among individuals.

### Clinical characteristics

**Figure 4:**
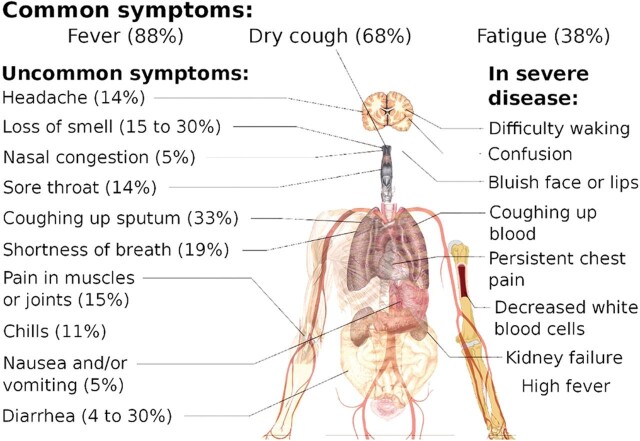
Common, uncommon and severe symptoms due to COVID-19.^18^

According to the report from WHO-China Joint Mission on COVID-19, 80% of infections are mild to moderate that includes pneumonia and non-pneumonia cases, 13.1% develop severe disease, while a further 6.1% develop critically serious disease requiring intensive care support.^17^ Typical presentation of symptomatic COVID-19 includes fever, cough and fatigue, though other less frequent and atypical symptoms have also been described. Symptoms of COVID-19 have been enumerated in figure 4.^18^ Severe COVID-19 has been defined as presence of tachypnea (≥30 breaths/min), oxygen saturation ≤93% at rest, >50% lung involvement on imaging or PaO2/FiO2 ratio <300 mm Hg, while critical disease has been defined as respiratory failure requiring mechanical ventilation, septic shock, or other organ dysfunction or failure requiring intensive care support.^19^ The risk of severe and critical disease is highest in the elderly (>60 years) and those with underlying conditions like diabetes, hypertension, obesity, malignancy, chronic respiratory disease, cardiovascular disease and liver disease.^20^ One study has suggested that children of all ages can get infected, and even though the majority of infections in children are asymptomatic or mild, outcomes can be poor in young children, especially infants with a 7–10% incidence of severe and critical disease.^21^ The global experience with COVID-19 since its outbreak has confirmed that no age is exempt from severe and critical disease requiring intensive care treatment and high mortality.

### Diagnosis

Laboratory findings associated with severe COVID-19 are decreased albumin, high C reactive protein, high lactate dehydrogenase, lymphopenia, eosinopenia, high erythrocyte sedimentation rate, leucopenia or leucocytosis, hyperbilirubinemia, elevated liver enzymes and high creatinine.^22^

Chest X-ray can show features of bilateral pneumonia (11–100%), unilateral pneumonia (1.5–85%), ground glass opacity (GGO) (13–100%) and acute respiratory distress syndrome (ARDS) in critical patients (17–67%). In early cases, chest X-ray may be normal, hence not considered sensitive enough to rule out COVID-19. Computed tomography (CT) of the chest shows positive findings in 84% patients, characteristically involving both lungs (lower zone more than upper zone and posterior area more than the anterior area).^23^ Characteristic CT features include GGO (40.3%), GGO with reticular pattern (62.9%), consolidation (33.9%), vacuolar sign (54.8%), microvascular dilation sign (45.2%), fibrotic streaks (56.5%), subpleural line (33.9%), subpleural transparent line (53.2%), air bronchogram (72.6%), bronchus distortion (17.7%), pleural thickening (48.4%), pleural retraction (56.5%) and pleural effusion (9.7%).^23^

For confirmation of the diagnosis of COVID-19, reverse transcription-PCR (RT-PCR) from oropharyngeal and nasopharyngeal swab is the current standard of testing, however, up to 30% false-negative rates may be observed with the current technique, especially in early cases. Chest CT alongside RT-PCR has been utilised for both screening and monitoring the clinical progress of patients. Early experience from the United Kingdom had shown greater sensitivity of chest CT compared with RT-PCR in the early stage of severe disease indicating CT abnormalities may appear before PCR positivity.^24^ However, RT-PCR from throat swab has remained the current standard for confirmation of the diagnosis of active infection in suspected patients. Serum antibody testing is another modality to detect IgM and IgG antibodies against SARS-CoV-2 for diagnosis and monitoring. IgM antibodies can be detectable 10 days after the onset of symptoms, while IgG antibodies can be detected after 12 days.^25^ This should, however, be interpreted with caution as seroprevalence could only mean a current or prior infection with the virus and further studies are needed to confirm if this translates to immunity preventing further re-infections.

### Severe and critical COVID-19

Even though 80% of COVID-19 cases are mild to moderate, the unlucky few who are more likely elderly or with underlying conditions go on to develop severe and critical disease with worsening symptoms as enumerated in figure 4.^18^ Characteristically, there is worsening of cough, fever and shortness of breath that would require hospital admission. Of those who get admitted in the hospital, 26–32% need admission in the ICU.^26^ Among those who develop severe disease, the median time to dyspnoea is 5–8 days, to development of ARDS is 8–12 days and ICU admission is between 10 and 12 days from the onset of symptoms.^26^ In comparison to the overall global mortality of 3.4%–7% from all confirmed cases (positive RT-PCR) worldwide, mortality among patients admitted in the ICU ranges from 39% to 72% between studies.^26^ This high mortality can be attributed to a combination of the severity of the disease with limited treatment options and excessively overburdened healthcare systems across the globe.^4 26^

Pathogenesis of severe and critical COVID-19 is complex and various theories have been proposed, most still undergoing active investigation. The most commonly accepted view is that SARS-CoV-2 attacks the ACE2 receptors in the lung that leads to intracellular viral replication until the cell bursts causing multiple virus particles to be released which then infects more cells. This also leads to activation of innate and adaptive immunity leading to harmful tissue damage, both locally and systemically due to cytokine storm. Patients with severe COVID-19 characteristically show features of dysregulated immune systemic response with drastically reduced numbers of CD4^+^ T cells, CD8^+^ T cells, B cells, natural killer cells, monocytes, eosinophils and basophils, along with an increase in neutrophil count.^27^ Elevated levels of pro-inflammatory cytokines including IL-6, IL-1beta, IL-2, IL-8, IL-17, G-CSF, GM-CSF, MCP1 and TNF are observed that characterises the cytokine storm.^28^ In the lungs, massive infiltration of neutrophils and macrophages occur promoting hyaline membrane deposition and diffuse alveolar damage, that leads to to respiratory failure and ARDS. Eventually, a pan-systemic damage to the heart, liver and kidney ensues due to widespread systemic inflammatory response leading to multi-organ dysfunction and immune-mediated damage. Even though outcomes are worse in the elderly, no age is exempt from severe disease.^21^ Furthermore, obesity has emerged as the single most important risk factor for hospital admission and worse critical care outcomes in patients less than 60 years of age.^29^

There are alternate hypotheses for the pathogenesis of severe and critical COVID-19 proposed in existing literature that needs validation with further research. According to one study, ACE2 receptor is expressed in significant quantities in the heart and this could explain symptoms of chest tightness and palpitations.^30^ Excessive myocardial damage, myocarditis and consequent cardiac failure have been proposed as a possible cause of death in severe COVID-19 disease as evidenced by a rise in cardiac-specific troponins during hospitalisation in 11.8% patients without pre-existing cardiac comorbidities who eventually died.^30^ Another study cited transient elevation of anti-phospholipid antibodies in critically ill patients due to SARS-CoV-2 viraemia as the cause of thrombotic events and disseminated intravascular coagulation.^31^ A neuroinvasive theory has also been proposed that SARS-CoV-2 has a potential to invade peripheral nerve terminals which then gains access to central nervous system including the brain stem via synapse-connected route and this neuroinvasive potential has been proposed to play a role in respiratory failure of severe COVID-19 patients.^32^ Another study proposes that SARS-CoV-2 attacks 1-beta chain of haemoglobin and captures the porphyrin to inhibit human heme metabolism, but this theory has been largely criticised due to its flawed methodology in interpreting the results.^33^

## TREATMENT OF COVID-19

Mild to moderate cases of COVID-19 can be managed expectantly with symptomatic therapy. Critical patients, however, need admission and their management involves supportive care and management of complications like pneumonia, respiratory failure, ARDS, septic shock, arrhythmias, cardiomyopathy, acute kidney injury, secondary bacterial infection, thromboembolism, gastrointestinal bleeding and critical illness polyneuropathy/myopathy.^26^ SARS-CoV-2, being primarily a respiratory pathogen, most patients who decompensate end up requiring respiratory support needing assisted ventilation, often with endotracheal intubation. One of the hallmarks of a pandemic of this proportion is that once the hospital capacity is overwhelmed, critical care outcomes are poor. The overwhelmingly high number of critical care admissions on a daily basis can potentially exceed the hospital capacity forcing clinicians to take life and death decisions based on triage. This was evidenced from the unfortunate experiences seen in USA and Italy where guidance were placed setting rules on who gets to live and who dies.^34^

### Pharmacotherapy

Many anecdotal reports have claimed effectiveness of various drugs such as tocilizumab (IL-6 receptor antagonist), chloroquine (antimalarial), hydroxychloroquine, azithromycin and antiviral drugs such as remdesivir, favipiravir and lopinavir/ritonavir and steroids.^35–37^ All of these drugs are being trialled in various combinations to identify an effective pharmacological regimen for the treatment of COVID-19.^38^

Some trials have already reported their results on the efficacy of a few of these drugs which have so far been dismal. The LOTUS trial on the efficacy of lopinavir-ritonavir on SARS-CoV-2 failed to show significant clinical improvement or reduction in mortality in patients with serious COVID-19.^39^ Similarly, a multinational registry analysis found no significant clinical benefit with chloroquine or hydroxychloroquine combinations with or without macrolide and in fact found decreased survival and higher incidence of ventricular arrhythmias in patients who received these drugs.^40^ Another trial from China on the efficacy of hydroxychloroquine on SARS-CoV-2 found no improvement in negative conversion rate but higher adverse events in patients who received the drug.^41^ The inefficacy of hydroxychloroquine/chloroquine with/without macrolide on SARS-CoV-2 shown in the reported studies has been an area of debate and controversy with ongoing claims of significant bias in the reporting of these results. A large randomised controlled trial on the effect of remdesivir found that it reduced median recovery time by 4 days and 14-day mortality from 11.9% to 7.1% and is one of the few drugs so far that has shown encouraging results until date.^42^ One of the largest trials testing a range of potentially different treatment options is the University of Oxford’s RECOVERY trial, the outcome of which is awaited, and which showed in a preliminary report that dexamethasone can reduce 28-day mortality by 35% in invasively ventilated patients and by 20% in patients on oxygen therapy without invasive ventilation.^43^ It is hoped that with more than 200 trials registered across the globe testing different drugs in varying combinations against SARS-CoV-2, an effective pharmacological therapy to COVID-19 would be discovered in future that could improve clinical outcomes.

## TRANSMISSION AND PREVENTION OF COVID-19

To understand the concept of prevention against COVID-19, it is important to understand the transmission of SARS-CoV-2. It must be emphasised that COVID-19 is a new disease and we are still learning about how it spreads. Evidence suggests that SARS-CoV-2 transmission to humans can occur by three methods—(a) Droplet spray in short range, (b) Fomite (contact) transmission and (c) Aerosol in long range.^44^ Droplet spray is generated when an infected person coughs, sneezes or even talks loudly and can be infective to others in close contact up to 6 feet(1.8 m). Some experts believe that this distance of 6 feet may not be safe enough a distance to prevent the spread of the virus.^45^ Often, the virus spreads through fomites when an infected person contaminates the object and gets picked up unwittingly by a new person who gets infected. According to a study on aerosol and surface stability of SARS-CoV-2 published in *New England Journal of Medicine*, SARS-CoV-2 remains viable in aerosols for at least 3 h, and stable on most objects like plastic (72 h), stainless steel (48 h), cardboard (24 h) and copper (4 h) for long durations.^46^ The reproduction number (R0), or the number of people catching the disease from one infected individual was initially thought to be between 2.2 to 2.7.^47^ However, emerging evidence based on the doubling time of 2.4 days during early phases of the epidemic suggests that SARS-CoV-2 could have been more contagious than initially estimated with an R0 value between 4.7 and 6.6.^48^ This could be attributed to the high number of asymptomatic and mild cases who can unknowingly transmit the infection.

Preventive strategies advocated at administrative level has varied between countries. WHO has repeatedly urged governments to pursue contact tracing as the backbone of COVID-19 response in every country. Countries that have worked hard on contact tracing in the early stages of the pandemic like China, Singapore, South Korea and Germany along with early lockdown of activities have shown a far lower death rate than countries that did not like the United Kingdom and USA.^49^ Self-isolation for 7 days if a person gets infected or 14 days if anyone in the family shows symptoms of infection has been suggested by the National Health Service, United Kingdom.^50^ Existing evidence, however, also shows that a period of 7–14 days may not be enough as patients can continue to shed the virus up till 37 days, as evidenced from a study in China that showed the mean duration of virus shedding to be 20 days.^51^ Washing hands with soap and water for at least 20 s, avoiding touching eyes, nose or mouth if the hands are not clean, frequent use of hand sanitisers, washing hands on return to home, covering of mouth and nose with tissue or sleeve while coughing or sneezing and immediate washing of hands afterwards has been recommended by the National Health Service to control the spread of infection.^52^ Few countries have also advised public to wear face masks or cover the mouth and nose with scarves and bandanas to prevent spread of infection, reserving respirators and medical masks for healthcare use.^53^ In spite these efforts, new infection rate was found to be rising at alarmingly high levels globally which is why the government of most countries had to impose a national lockdown of unnecessary activities to prevent community spread of infection with the aim to reduce R0 to less than 1.

It has been suggested by most authorities that the real victory over COVID-19 can be achieved by mass vaccination that would produce herd immunity in the population. There is an ongoing speculation on when a safe vaccine would be ready for mass administration, with some authorities suggesting that it may take up until 18 months while others claiming it would be available by the autumn of 2020. More than 100 trials are ongoing in different countries, out of which the USA, United Kingdom and China are among the few countries to began with human trials. In the United Kingdom, the University of Oxford’s Jenner Institute and Oxford Vaccine Group have begun recruitment for a human trial on the vaccine, ChAdOx1 nCoV-19, which is based on a harmless chimpanzee adenovirus genetically engineered to contain the S-protein of SARS-CoV-2 [ClinicalTrials.gov Identifier: NCT04324606].^54^

Another potential therapy that is being investigated in trials is the effect of convalescent plasma of patients who have recovered from SARS-CoV-2 infection. This is based on a few studies that showed promising results with convalescent plasma containing anti-COVID antibodies when transfused to patients suffering from severe COVID-19.^55^

It is very difficult to predict the direction of the SARS-CoV-2 pandemic. It remains to be seen if the disease will largely disappear like SARS-CoV and MERS-CoV or if it will continue to exist in the community with repeated outbreaks in future. The global health community is dealing with the brunt of the pandemic and many healthcare workers have sacrificed their lives treating patients with COVID-19. More than a hundred healthcare workers have died in this pandemic in the United Kingdom.^56^ According to a few countries, 5–15% of all reported infections have been among healthcare workers caring for these patients.^57^ Many of these healthcare workers have caught the infection in the line of duty due to shortages of personal protective equipment (PPE) due to under-preparedness by different governments in anticipating the crisis, often paying the price with their lives. Once the peak has passed and flattening of the curve is achieved, the number of new cases and deaths are expected to reduce in numbers. However, the real chance of preventing further peaks and future outbreaks is essentially going to require the development of an effective antiviral strategy like vaccines (preventive) and drugs (therapeutic) that can act on SARS-CoV-2, hence reducing the clinical sequelae of an infection and this is something which the scientific community worldwide should focus on. Being prepared for another future pandemic of this proportion is also worth mentioning and prompt action by the government in early phases of the transmission with contact tracing along with stockpiling necessary PPE in advance for the healthcare workers could help contain the pandemic with far lesser consequences than COVID-19.

**Main messages**

- SARS-CoV-2 is the cause of a serious life-threatening disease known as COVID-19.
- Typical symptoms of COVID-19 include fever, cough, shortness of breath and fatigue, while some atypical symptoms have also been described.
- 80% of infections are mild to moderate that includes pneumonia and non-pneumonia cases, 13.1% develop severe disease, while a further 6.1% develop critically serious disease requiring intensive care.
- Treatment is primarily supportive, and involves critical care input to maintain major organ functions throughout the course of the illness.
- Prognosis is dismal in patients with severe disease requiring invasive ventilation.
- Trials are ongoing in a quest for antiviral strategies like drugs and vaccines.

**Current research questions**

- Is there a role of genetics in the prognosis of COVID-19?
- Will the disease disappear after the COVID-19 pandemic is over or will it continue to live in the community causing repeated outbreaks?
- Will an effective and safe vaccine to prevent COVID-19 be discovered in future and when?
- Will an effective and safe antiviral drug regimen be discovered in future to either treat or prevent progression to severe disease in COVID-19?
- How can the world be better prepared for another pandemic of this magnitude in future?

**Self-assessment questions**

1. Which of the following is true of COVID-19?
  - It is caused by the SARS-CoV-2 virus.
  - It was first identified in Italy.
  - Genetic analysis of the virus shows a structural resemblance to bat coronavirus.
  - The disease primarily affects the kidneys with the respiratory system affected as the second most common organ system.
2. Severe COVID-19 is characterised by—
  - Respiratory rate of >30 breaths per minute.
  - Prognosis is worse in children compared to age >60 years.
  - Prognosis is worse in patients with BMI <35 compared to BMI >35.
  - Loss of smell and taste suggests severe disease.
3. Diagnostic criteria for COVID-19 includes—
  - Antibody ELISA is the gold standard test for diagnostic confirmation of COVID-19.
  - Chest X-ray is confirmatory is almost all positive cases.
  - CT chest can show features of ground glass opacity, consolidation and fibrosis.
  - Lymphopenia alongside neutrophilia is characteristic.
4. Regarding treatment of COVID-19—
  - Critical patients can be successfully treated with hydroxychloroquine in all cases.
  - Invasive ventilation can be viewed as a good prognostic sign due to better respiratory support.
  - LOTUS trial failed to show any significant clinical improvement with lopinavir-ritonavir combination.
  - RECOVERY trial testing a range of potentially different treatment options is a major drug trial on COVID-19 in the United States of America.
5. Transmission of COVID-19 can be reduced by—
  - Frequent hand washing with soap.
  - Maintaining sufficient distance from other people (social distancing).
  - Prophylactic antibiotics.
  - Visiting crowded areas like supermarkets and gym instead of open spaces like park.

**Answers**

1. A—True, B— False, C—True, D—False.
2. A—True, B— False, C—False, D—False.
3. A—False, B— False, C—True, D—True.
4. A—False, B— False, C—True, D—False.
5. A—True, B— True, C—False, D—False.

**Key references**

- ‘Coronavirus Pandemic (COVID-19)’. Our World In Data. Available at https://ourworldindata.org/coronavirus (Accessed on 30 May 2020).
- Zhou, P., Yang, X., Wang, X. *et al.* A pneumonia outbreak associated with a new coronavirus of probable bat origin. *Nature*. 2020;579:270–73.
- Guan WJ, Ni ZY, Hu Y, *et al.* Clinical characteristics of coronavirus disease 2019 in China. *N Engl J Med.* 28 February 2020: NEJMoa2002032.
- Beigel JH, Tomashek KM, Dodd LE, *et al.* Remdesivir for the treatment of COVID-19**—**preliminary Report. *N Engl J Med*. 22 May 2020.
- ClinicalTrials.gov. A Study of a Candidate COVID-19 Vaccine (COV001). Identification No. NCT04324606. Available at https://clinicaltrials.gov/ct2/show/NCT04324606 (Accessed on 30 May 2020).
